# Correction: Cytokine-induced chromatin accessibility in whole blood neutrophils links to sepsis transcriptional states

**DOI:** 10.3389/fimmu.2026.1925697

**Published:** 2026-07-23

**Authors:** Justin Cayford, Brandi Atteberry, Akanksha Singh-Taylor, Andrew Retter, Benjamin P. Berman, Theresa K. Kelly

**Affiliations:** 1 Innovation Lab, Volition America, Carlsbad, CA, United States; 2 Department of Developmental Biology and Cancer Research, The Hebrew University of Jerusalem, Jerusalem, Israel

**Keywords:** ATAC-seq, chromatin structure, cytokine storm, innate immune system, NETosis, neutrophil extracellular traps, NETs

There was a mistake in **Figure 4D** as published. The y-axes were not correctly updated when adjusting the size of the figure as all are 12.5 and at the same position, regardless of the y-axis tick marks. The original version had the correct values and were used for correcting this copy/paste error. The corrected **Figure 4D** appears below.

**Figure 4:**
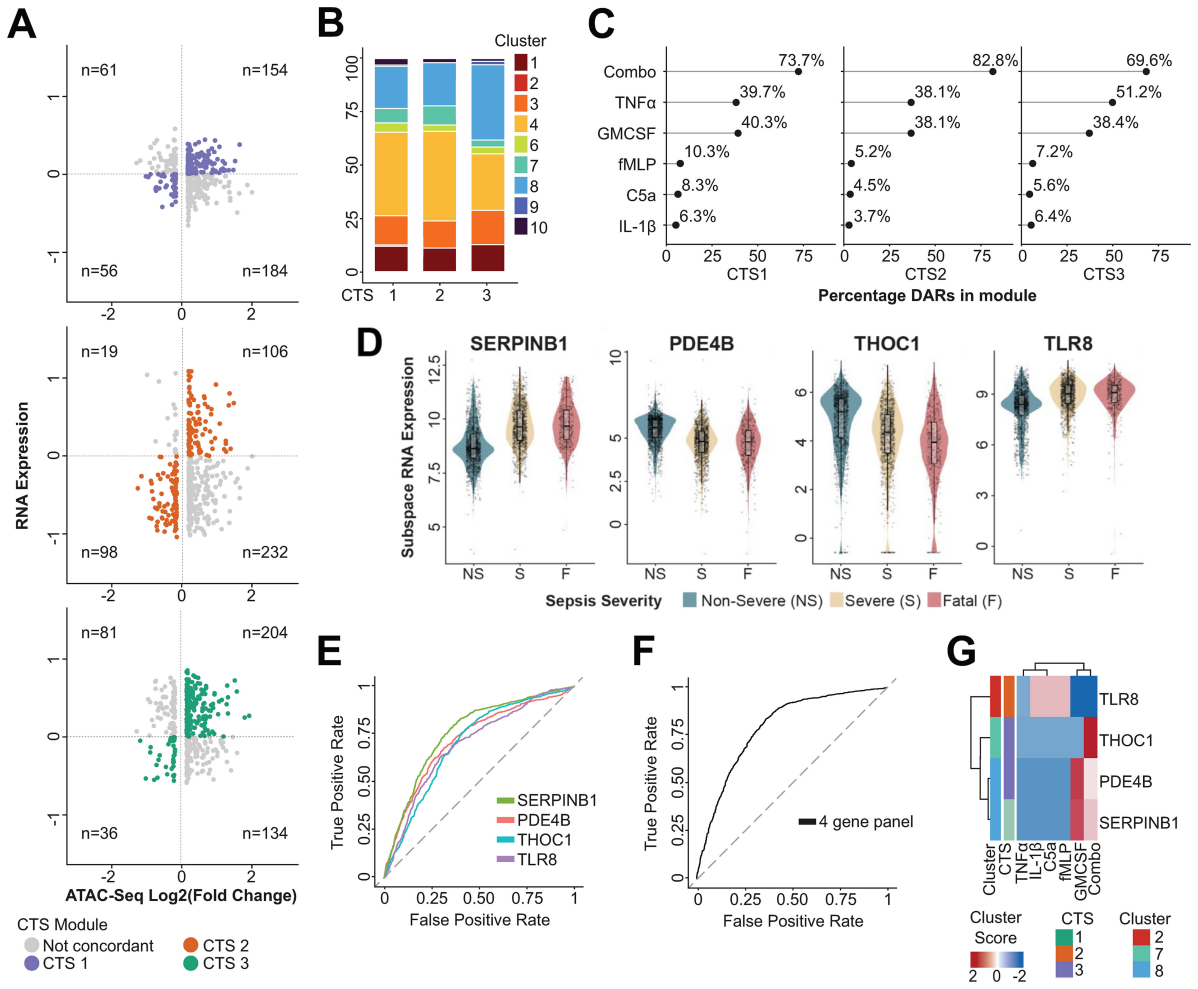
Chromatin accessibility signatures associated with transcriptional programs linked to progression of severe disease: **(A)** Scatterplots comparing promoter-associated chromatin accessibility changes to mean gene expression values (across donors) from the VANISH cohort. Genes are grouped by CTS module and colored by concordant directionality between accessibility and transcription. Top – CTS1; green points indicate concordance – n=204 for up/up and n=36 for down/down. Middle – CTS2; orange points indicate concordance – n=106 for up/up and n=96 for down/down. Bottom – CTS3; purple points indicate concordance – n=154 for up/up and n=56 for down/down. **(B)** Distribution of ATAC cluster assignments among concordant promoter-DARs for each CTS module. Bars represent the proportion of regions assigned to each chromatin cluster, highlighting shared and distinct regulatory composition. For cluster assignment colors: 1 – dark blue, 2 - blue, 3 - light blue, 4 – teal, 5 – green, 6 – light green, 7 – light orange, 8 – orange, 9 – red, 10 – dark red. **(C)** Lollipop plots summarizing the relative contribution of individual stimulation conditions to CTS associated promoter accessibility changes. For each CTS module, the percentage of regions showing differential accessibility is shown. Combo, TNF-a, and GM-CSF account for the majority of response across CTS modules. **(D)** Expression of ATAC-linked genes in the SUBSPACE cohort across sepsis severity grades (Green – non-severe, yellow – severe, and red – fatal). Violin and boxplots show the distribution of normalized gene expression for the four genes (SERPINB1, PDE4B, THOC1, and TLR8) retained for downstream analysis. Individual points represent individual donors (non-severe: n=1,293, severe: n=1,203, and fatal: n=387). **(E)** Receiver operating characteristic (ROC) curves evaluating the ability of genes to distinguish severe/fatal from non-severe disease in the SUBSPACE cohort. Four genes all contained an area under the curve (AUC) > 0.70 (Green - SERPINB1; AUC = 0.76, Red - PDE4B; AUC = 0.72, Blue - THOC1; AUC = 0.7, and Purple - TLR8; AUC = 0.7). **(F)** Similar to **(E)** but ROC curve for all four genes (SERPINB1, PDE4B, THOC1, and TLR8) combined (AUC = 0.79). **(G)** Heatmap of promoter-associated chromatin accessibility for the four genes across all stimulation conditions. Rows represent the promoter-associated peak per gene, and columns represent the stimulation compared to vehicle. Values are z-score normalized across the comparisons. Row annotations indicate ATAC cluster assignment and CTS module (CTS1 – green, CTS2 – orange, CTS3 – purple).

The original version of this article has been updated.

